# Immunization data quality and decision making in pertussis outbreak management in southern Ethiopia: a cross sectional study

**DOI:** 10.1186/s13690-022-00805-6

**Published:** 2022-02-14

**Authors:** Mesele Damte Argaw, Binyam Fekadu Desta, Zergu Taffesse Tsegaye, Aychiluhim Damtew Mitiku, Afework Ayele Atsa, Bekele Belayihun Tefera, Deirdre Rogers, Ephrem Teferi, Wondwosen Shiferaw Abera, Ismael Ali Beshir, Zelalem Abera Kora, Sisay Setegn, Amare Assefa Anara, Tadelech Sinamo, Rudzani Muloiwa

**Affiliations:** 1 USAID Transform: Primary Health Care Project, JSI Research & Training Institute, Inc, Addis Ababa, Ethiopia; 2 Daro Malo Woreda Health Office, Dara Malo, Ethiopia; 3 USAID Transform: Primary Health Care Project, Pathfinder International, Addis Ababa, Ethiopia; 4 JSI Research & Training Institute, Inc. Boston, Boston, USA; 5 Department of Paediatrics & Child Health, Groote Schuur Hospital, University of Cape Town, Cape Town, South Africa

**Keywords:** Accuracy, Consistency, Completeness, Timeliness, Quality Index, Expanded Program of Immunization, Dara Malo, Southern Ethiopia

## Abstract

**Background:**

The aim of this study was to investigate the quality of immunization data and monitoring systems in the Dara Malo District (Woreda) of the Gamo Administrative Zone, within the Southern Nations, Nationalities, and Peoples’ Region (SNNPR) of Ethiopia.

**Methods:**

A cross-sectional study was conducted from August 4 to September 27, 2019, in Dara Malo District. The district was purposively selected during the management of a pertussis outbreak, based on a hypothesis of ‘there is no difference in reported and recounted immunization status of children 7 to 23 months in Dara Malo District of Ethiopia’. The study used the World Health Organization (WHO) recommended Data Quality Self-Assessment (DQS) tools. The accuracy ratio was determined using data from routine Expanded Program of Immunization (EPI) and household surveys. Facility data spanning the course of 336 months were abstracted from EPI registers, tally sheets, and monthly routine reports. In addition, household surveys collected data from caretakers, immunization cards, or oral reports. Trained DQS assessors collected the data to explore the quality of monitoring systems at health posts, health centers, and at the district health office level. A quality index (QI) and proportions of completeness, timeliness, and accuracy ratio of the first and third doses of pentavalent vaccines and the first dose of measles-containing vaccines (MCV) were formulated.

**Results:**

In this study, facility data spanning 336 months were extracted. In addition, 595 children aged 7 to 23 months, with a response rate of 94.3% were assessed and compared for immunization status, using register and immunization cards or caretakers’ oral reports through the household survey. At the district level, the proportion of the re-counted vaccination data on EPI registers for first dose pentavalent was 95.20%, three doses of pentavalent were 104.2% and the first dose of measles was 98.6%. However, the ratio of vaccination data compared using tallies against the reports showed evidence of overreporting with 50.8%, 45.1%, and 46.5% for first pentavalent, third pentavalent, and the first dose of measles vaccinations, respectively. The completeness of the third dose of pentavalent vaccinations was 95.3%, 95.6%, and 100.0% at health posts, health centers, and the district health office, respectively. The timeliness of the immunization reports was 56.5% and 64.6% at health posts and health centers, respectively, while the district health office does not have timely submitted on time to the next higher level for twelve months. The QI scores ranged between 61.0% and 80.5% for all five categories, namely, 73.0% for recording, 71.4% for archiving and reporting, 70.4% for demographic information, 69.7% for core outputs, and 70.4% for data use and were assessed as suboptimal at all levels. The district health office had an emergency preparedness plan. However, pertussis was not on the list of anticipated outbreaks.

**Conclusion:**

Immunization data completeness was found to be optimal. However, in the study area, the accuracy, consistency, timeliness, and quality of the monitoring system were found to be suboptimal. Therefore, poor data quality has led to incorrect decision making during the reported pertussis outbreak management. Availing essential supplies, including tally sheets, monitoring charts, and stock management tools, should be prioritized in Daro Malo District. Enhancing the capacity of healthcare providers on planning, recording, archiving, and reporting, analyzing, and using immunization data for evidence-based decision making is recommended. Improving the availability of recording and reporting tools is also likely to enhance the data accuracy and completeness of the community health information system. Adapting pertussis outbreak management guidelines and conducting regular data quality assessments with knowledge sharing events to all stakeholders is recommended.

**Supplementary Information:**

The online version contains supplementary material available at 10.1186/s13690-022-00805-6.

## Background

Immunization is recognized as one of the most successful and cost-effective public health interventions to ensure the healthy lives of children [1]. Every year, vaccines save 2–3 million lives, while millions more are protected from disease and disability [1]. Immunization is one of the best buys in global health strategies and has contributed to Ethiopia achieving Millennium Development Goal 4 (MDG 4) three years ahead of its target, and reducing its child mortality rate by 23% [2]. Immunization improvements also contribute to the achievement of the sustainable development goals (SDGs) [3]. Ethiopia recognizes the crucial role immunization plays in child health and affirms its responsibility to ensure that every child is protected from vaccine-preventable diseases.

In 2017, approximately 85% of infants worldwide received three doses of pentavalent vaccine (Diphtheria, Pertussis, Tetanus, Hepatitis B, and Hemophilus Influenzae type b). However, notwithstanding the progress that has been made, lower-middle-income countries continue to encounter the substantial burden of vaccine-preventable diseases, and their vaccination coverage is lagging behind that of upper-middle-income countries [4], leaving 19.9 million children vulnerable to vaccine-preventable diseases [5]. Among those, approximately 60% are in 10 developing countries, including Ethiopia [6].

Studies have found a high burden of vaccine-preventable diseases among vaccinated children due to the low vaccination coverage rates, inappropriate timing of vaccinations, and inadequate potency of vaccines [7]. The average three doses of pentavalent vaccine coverage in low-income countries were 15 percentage points lower than that of high-income countries in 2011 [8]. Based on 2017 WHO and UNICEF estimates of national immunization coverage, the three doses of pentavalent vaccination in Africa were 72% [6].

The Expanded Program of Immunization (EPI) in Ethiopia, which was launched in 1980, has shown steady progress in increasing coverage for all antigens [9]. Additional vaccines beyond the traditional vaccines, Hib and Hep-B in 2007, PCV in 2011, and MCV2 in 2019, were included in the routine immunization program. Between age of 45 to 105 days, an infant is eligible to receive all the three doses of pentavalent vaccines (Additional file 1) [9]. In 2017 and 2018, the study area of Dara Malo district, reported 103.0% and 94.8% for three doses of pentavalent vaccination rates, respectively [10]. Despite the reported high immunization coverage, from August 2018 through to January 2019, there was an outbreak of whooping cough, with 1,840 cases reported and six subsequent deaths.

In line with international health regulations, every district of Ethiopia is expected to adapt and establish a nationally endorsed public health emergency management (PHEM) system. The principles of PHEM consist of addressing emergency preparedness, early detection, response, and recovery for adverse effects of reported diseases. Well-functioning, progressive emergency preparedness is expected to anticipate future emergencies, take preventive and preparatory measures, and build disaster-resistant and disaster-resilient communities. In addition, it should guide the implementation of risk-driven emergency management where district health systems identify health hazards, and analyze risks and impact regularly using health data collected through the community-based surveillance system. Health workers are mandated to use evidence for decision making on priorities and resource allocation. The third step of outbreak management is early detection and response, which consists of initiating epidemic investigation activity within 24 h after receiving reports on the occurrence of an outbreak, confirming the existence of the outbreak, and initiating implementation of prompt responses. The fourth step of outbreak management deals with the implementation of short and midterm recovery interventions from adverse social and economic future impact. At this stage, the health system should continue its management lasting over two months by identifying the extent of the damage caused by an outbreak, providing the support needed for recovery and restoration, monitoring the health situation of affected communities, and promoting healthy behavior.

The outbreak investigation team hypothesized that data inaccuracy and poor quality of monitoring systems were the most likely reasons for the occurrence of the outbreak. In addition, the timeliness and completeness of routine immunization data might affect the decisions of public health emergency managers. The team also observed discrepancies between the reported data and household survey coverages [11]. Therefore, conducting DQS would have helped to strengthen the community-based surveillance system and identify activities that can improve routine EPI services in the district [12]. This study aims to investigate the quality of immunization data, monitoring systems, and outbreak management practices in the Dara Malo District of the Gamo Administrative Zone, within the SNNP Region of Ethiopia.

## Methods

### Study area and population

Dara Malo is one of 160 districts administered under the SNNP Region of Ethiopia. The district has 24 of the lowest administrative structures, which consist of one urban village and 23 rural villages [10]. Based on the 2007 Ethiopian national census, the projected population of the Dara Malo District in 2018 was 107,715. From the total population, 16,814 (15.6%) were children under five years old, and an estimated 3,436 (3.19%) were under the age of one [10]. However, the district’s 23 health posts and four health centers only offer outreach immunization services once a month. From July 2018 to June 2019, the reported third dose pentavalent and measles vaccination coverages were 94.8% and 94.0%, respectively. Fourteen, (60.8%) villages (kebeles) where 69,587 people live within the district were affected by pertussis and measles outbreaks [11].

### Study design and sampling methods

A cross-sectional study was conducted from August 4 to September 27, 2019, in the Dara Malo District, Gamo Administrative Zone, southern Ethiopia. The facility-based survey collected data from 23 health posts, four health centers, and a district health office. The household data were collected from sampled households. The sample size was determined using a single population proportion formula with the assumptions of 53% proportion of third dose pentavalent vaccination coverage [9], a 5% margin of error, with a 95% confidence interval, and a 10% non-response rate [12]. The calculated sample size was 631.

### Data collection and analysis

The WHO’s (2005) recommended self-assessment tools were customized for this study (Additional file 2) [13]. The tools were adapted to capture the accuracy, completeness, and timeliness of routine immunization data from different sources; namely, registers, tally sheets, household surveys, and reports (Additional file 3). In addition, data quality monitoring tools helped to capture the availability of essential supplies such as vaccines, diluents, syringes, vaccination cards, tally sheets, and registers.

The data were collected by twelve trained health professionals with Bachelor of Sciences (BSc.) level of educational achievement, who also had experience of document reviews. In addition, two trained health workers with master’s degrees in public health oversaw the quality of the collected data.

The two-day training was facilitated for data collectors and supervisors on the objectives of the study, data collection methods, and ethical issues. Collected data were checked for completeness and consistency on a daily basis, and data were double entered for consistency.

Facility-based survey data on first and third doses of the pentavalent and first dose of measles-containing vaccines were reviewed from July 2018 through to June 2019. In addition, trained assessors evaluated the data quality monitoring system using WHO’s (2005) DQS tools [13]. Finally, household surveys were conducted to collect primary data on immunization status for children aged between 7 and 23 months.

The data were entered into a spreadsheet, cleaned, summarized, and analyzed using Microsoft Excel® and Statistical Packaged for Social Sciences Research (SPSS IBM V 20) [14]. The accuracy ratio, proportions of completeness, and timeliness were calculated. The definitions and assumptions are presented below.

#### Accuracy ratio

Based on the WHO’s definition, data accuracy is the ratio between the number of vaccinations verified or re-counted from a source at one level (numerator) compared to the number of vaccinations reported by that level to more central levels (denominator) [13]. In this study, first and third doses of pentavalent and the first dose of measles vaccination recounted from the register, tally sheet, and caretakers’ report during the household survey were taken as a numerator, and report of health posts, health centers, and woreda health office then submitted to the next higher level were considered denominators. To characterize the reporting accuracy, national recommended categories were used, i.e. consistent if the ratio reported was ≥ 90.0 and < 110.0%; overreported if it was < 90.0%, and underreported if the ratio was ≥ 110.0% [14]. In addition, the overreporting health entities were further classified with reasons for inaccuracies, namely, missing tally sheets, discrepant recounted tally sheets against reports, and both missed and/or discrepant reports [15].

#### Completeness

According to WHO’s definition data completeness is a percentage with the number of reports received in the numerator and the number of reports expected during a period of time as a denominator [13]. In this study, the number of the health posts, health centers, and woreda health office reports received at the next higher-level health system tier for 12 months (July 2018 – June 2019) was computed against the number of expected reports [15].

#### Timeliness

Defined by the WHO as a percentage with the number of reports that were received on time (using a deadline set by the EPI office) were the numerator and the number of reports expected during a period of time were the denominator [13]. In this study timeliness was considered for health post, health center, and woreda health posts, if there was evidence of written date at least once in each report in line with the national health information management system recommendation received before 22^nd^ – 23^rd^ and 26^th^ day of each month at the next reporting level for 12 months (July 2018 – June 2019) were considered to evaluate timeliness at each level [15].

#### Quality index

The WHO’s (2005) DQS tools have 45 elements of variables for health facilities and 51 elements of variables for a district health office. These tools were adapted for this study and assessed by trained data collectors on the quality of the immunization monitoring system [12]. The variables were categorized into five; namely, recording; archiving, and reporting; demographics; core outputs or analysis and evidence of use of data for action. Trained data collectors provided scores for each question. A “no” response scored “0”; and “yes” response was scored from “1 to 3” based on its importance, and a “NA” response was not considered in the denominator. The QI was calculated after summing up all scores of evaluators and divided by the sum of possible maximum scores in five categories [12].

## Results

### Accuracy ratio

**Fig. 1:**
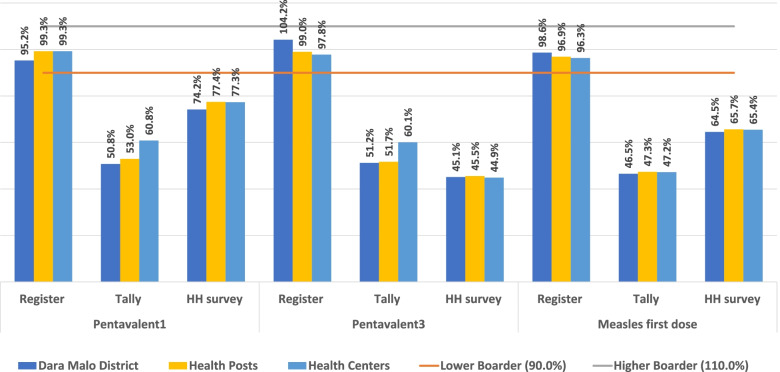
Consistency of immunization data that were reported and recounted at health posts, health centers and district health offices over one year, July 2018 – June 2019. The bar chart clearly depicts that the report and register data were consistent as per the national recommended < 110.0% and ≥ 90.0% and the high level of overreporting between reports compared with household survey and recounted tallies

Data from three hundred and thirty-six facility months were extracted. In addition, 595 children aged 7—23 months, with a response rate of 94.3%, were assessed and compared for immunization status between register and immunization card or through caretakers’ oral reports on household surveys. At the district level, the proportion of the re-counted vaccination data on EPI registers for the first dose of pentavalent was 95.2%, 104.2% for the third dose, and 98.6% for measles (Fig. 1). The ratio of vaccination data compared using tallies against the reports had evidence of overreporting, which were 50.8%, 45.1%, and 46.5% for first pentavalent, third pentavalent, and the first dose of measles vaccination, respectively. Eighteen (64.3%) facilities were classified having as discrepantly recounted, and the remaining 10 (37.3%) were both those missing data and discrepantly recounted facilities (Fig. 1).

### Completeness and timeliness of the reporting

Table 1 illustrates the completeness and timeliness of reports on the third dose of the pentavalent and first dose of measles at primary health care entities. The completeness of both the third dose of pentavalent and measles ranged from 95.3% to 100.0%. The lowest completeness report was for health posts at 95.3%, and the highest completeness percentage was 100.0% for district health offices. The timeliness of immunization reports ranges from the lowest 0.0% for the district health office, followed by 56.5% for the health posts, and the highest was 66.4% for health centers.

**Table 1:** Completeness and timeliness of immunization reports by reporting primary healthcare entities, 2019

Reporting facility	Completeness of reports (%)		Timeliness of reports (%)	
	Pentavalent (3rd dose)	Measles	Pentavalent (3rd dose)	Measles
**Health posts**	95.3%	95.7%	56.5%	56.5%
**Health centers**	95.6%	95.8%	64.6%	64.6%
**District health office**	100.0%	100.0%	0.0%	0.0%

The table presents the proportion of completeness and timeliness of routine immunization reports by three levels, i.e., health posts, health centers and district health offices

### Quality of the monitoring system

**Fig. 2:**
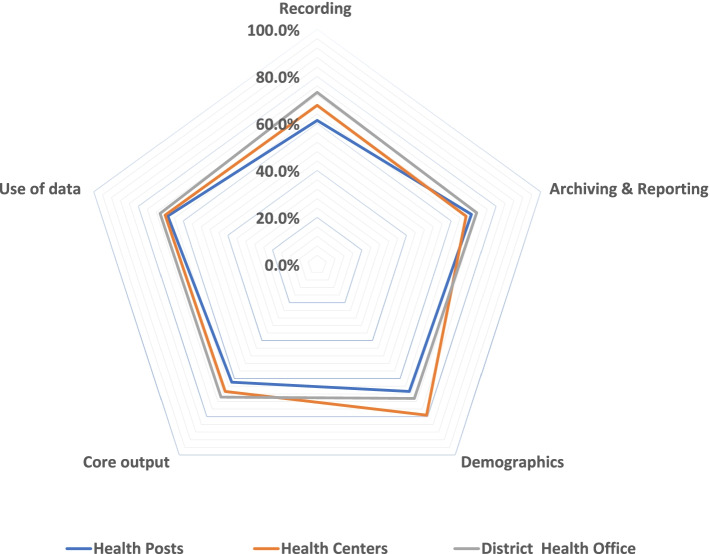
Quality indices for five categories of quality monitoring systems at health posts, health centers and district health offices, September 2019. The radar chart clearly shows that the quality of the monitoring system in all five categories was sub-optimal at the health post, health center and district health office levels

Table 2 and Fig. 2 illustrate the quality index (QI) scores using five major broad categories: recording, archiving and reporting, demographics, core output, and data use. The lowest QI score was 61.0% for recording at the health post, and the highest QI was 79.2% for demographic information at health centers. The quality of the monitoring system for the district health office ranges from 69.7% on core outputs or analysis to 73.3% for the recording category.

**Table 2:** Quality Index (QI) for the five components of a monitoring system at the health posts, health centers and district health office, 2019

Name of cluster		Recording	Archiving & reporting	Demographics	Core output	Data use
Wacha	Maximum score	270	234	72	126	108
	Acquired score	154	171	47	79	69
	QI	57.0%	73.1%	65.3%	62.7%	63.9%
Dara Dime	Maximum score	225	195	60	105	90
	Acquired score	153	157	40	57	56
	QI	68.0%	80.5%	66.7%	54.3%	62.2%
Shela Deda	Maximum score	225	195	60	105	90
	Acquired score	129	112	40	63	60
	QI	57.3%	57.40%	66.7%	60.0%	66.7%
Bobe Noyere	Maximum score	315	273	84	147	126
	Acquired score	199	180	57	100	92
	QI	63.2%	65.9%	67.9%	68.0%	73.0%
Health Posts	Maximum score	1035	897	276	483	414
	Acquired score	635	620	184	299	277
Health Centers	Maximum score	180	156	48	84	72
	Acquired score	122	104	38	56	49
District Health Office	Maximum score	15	42	27	33	27
	Acquired score	11	30	19	23	19

The table depicts maximum scores, acquired scores, quality indices by cluster and primary health care entities

### Outbreak management

Though pertussis was not mentioned in the anticipated disease outbreak list of Daro Malo District, the health officials had a 2,550 USD budgeted emergency preparedness plan to address measles and malaria for the year 2018/19. On September 1^st^, 2018, a child with a history of sever coughing during at night for more than three weeks was identified as an index case. In addition, the district health office received reports of unusual health conditions in the neighbors of the index case. A rapid response team (RRT) was established, capacitated, and deployed to the affected villages within 48 h. The RRT consisted of six people including the district PHEM focal person, a public health officer, a nurse, a laboratory technician, and health extension workers. The RRT assessed the signs and symptoms of the suspected cases and defined probable pneumonia cases as they witnessed coughs plus mild fever and fast paced breathing. From the 4th – 13th September 2018, the team identified a total of 471 pneumonia suspected cases. These cases were treated with two antibiotics, namely, amoxicillin and cotrimoxazole, for seven days. Simultaneously, the line list of cases and weekly surveillance reports were shared with the Ethiopian Public Health Institute, the World Health Organization, and USAID-funded development partners. In addition, the district health office requested its development partners’ technical, financial, and drugs assistances.

After two months, with the support of field epidemiologists the outbreak was reassessed, and the possible cause was redefined to probable pertussis infections. Then, pertussis suspected cases were defined as non-improving coughing lasting for 14 days or more, coughing of any duration with paroxysms, or coughing of any duration with a whoop. To confirm the diagnosis, 50 samples of 25 pertussis suspected patients were collected from nasopharynx using Dacron swabs (COPAN, Floq Technologies, Brescia, Italy) and transported in a cold box with ice packs to a central national laboratory. The outbreak was confirmed with nine Polymerase Chain Reaction (PCR) for *Bordetella pertussis,* and *Bordetella para-pertussis* (Real Star® Bordetella PCR Kit 1.0. Altona Diagnostics) positive samples. Then, with active case identification, an additional 1,369 were confirmed, and probable cases were treated with macrolides (i.e., erythromycin 40 mg/kg/day divided in 3 doses for 10 days and azithromycin 10 mg/kg on day 1 and 5 mg/kg on days 2–5 as a single dose). In addition, almost all (69,587) residents living in 14 villages of the district were covered with a prophylactic five-day mass drug administration using azithromycin observed therapy. After controlling the outbreak, the RRT assessed and organized supplementary vaccination campaigns. In January 2019, a total of 7,548 doses of pentavalent vaccination were given to all defaulters and eligible children aged less than two years.

## Discussion

This study evaluated the immunization data accuracy and quality of the monitoring system using one-year data extracted from 23 health posts, four health centers, and a district health office. In addition, the study revealed the presence of sub-optimal outbreak management practices. The results have provided objective evidence showing the existence of wide differences between reported and recounted immunization status of children in the Daro Malo District. EPI data used to assess the performance of the Dara Malo District were found to have poor accuracy, consistency, completeness, and timeliness. This might have led the health system to misunderstand the quality of services, as the district health system lacks the capacity to effectively use the quality of the immunization monitoring system for evidence-based timely decision making and response to vaccine-preventable disease outbreaks [16–18]. Hence, the most likely reason for delayed identification, verification, and control measures might be related to the poor immunization data quality and lack of outbreak management capacity at the district health system level [19].

Data verification mainly helps us to ensure quality data for evidence-based practices and quality of service delivery at the primary health care level. The accuracy ratio calculated between routine immunization reports and evidence of hand-written information on the EPI register was consistent with the Ethiopian national recommended good quality category [15]. However, there were wide discrepancies between routine immunization reports, tallies, and household survey data. The health centers and their satellite health posts are the source of overreporting within the district health system. The lowest overreporting was revealed as 60.8% for the first dose of pentavalent vaccination, and the highest overreporting was 44.9% for the third dose of pentavalent, which was documented in health centers. Similarly, overreporting was observed at health posts and at the district health office level. The high level of overreporting might have occurred due to a lack of supplies. This was consistent with the observed availability of tally sheets in two-thirds of primary healthcare entities (64.3%;18/28). This finding was much higher than the reported poor data concordance between child registers and facility tally sheets [20–23]. There should be efforts to improve the poor data accuracy through conducting regular data quality assessment and dissemination of findings to all stakeholders. In Sierra Leone, disease surveillance data accuracy was significantly improved after dissemination of baseline assessments [24]. Similar, Tijani et al. (2021) confer that data accuracy and completeness was improved through an established digitalized health information system in Nigeria [25]

The timeliness of immunization reports ranged from zero percent at a district health office, to 64.6% for health centers. This finding was in line with the observed schedule for arranged outreach immunization services, which overlaps with the reporting deadline schedule in all 12 months. Since there were no static immunization services in the district, the health office prolongs the reporting period to include the most recent activities until the health posts and health centers complete outreach services and submit their latest reports. This finding was in line with a 70% overall timeliness report of Kebede et al. (2020) in the western part of Ethiopia [26]. In addition, in a north-western province of Ethiopia, a qualitative study documented that the reason for low-levels of timeliness of reports was due to health workers not being familiar with routine health system reporting deadlines [27].

Completeness of immunization reports requires the availability of the reports with the necessary information in the required fields [27]. The completeness of immunization reports ranged from 95.0% to 100.0%. The results showed that almost all reports during the study period were available in the next highest reporting level of primary healthcare entity. This finding was consistent with the high rate of completeness reported in Iran [28].

The study shows that in the health posts, health centers, and in the district health office, there are problems with data recording, analyzing, and use of data for evidence-based decision making at the point of data production. The results of QI in all five major categories, namely, demographic information, recording, archiving and reporting, core outputs (analysis), and use for data for evidence-based decisions, were suboptimal at all primary healthcare entities. This could be explained by a number of influencing factors, to mention some: lack of capacity and skills to improve performance of essential health services, poor technical support, lack of verification, motivation, and rewarding mechanisms at the system level. This finding was consistent with the quality index of 55.0% reported by Tunisa by Chahed et al. (2013) [29]. Similarly, Yawson et al. (2017) confer that in Ghana, more than two-thirds of districts were challenged to achieve effective coverage [30]; Machingaidze et al. (2013) [21] and Bosch-Capblanch et al. (2009) identified a shortage of skilled manpower as affecting the strength of the immunization system in Africa and 41 low-income countries, respectively [31]. However, the results of the facility and community-based survey revealed that there are areas that need improvement at the primary healthcare entity level in terms of accuracy, timeliness, and immunization monitoring system.

The district health office had an emergency preparedness plan. However, pertussis was not on the list of anticipated outbreaks. Organizing district level rapid response team and deployment to the affected community within two days was in line with the national PHEM guidelines. However, the RRT had limited capacity and missed the most likely causes of the outbreak. This might be related to the non-specific nature of the sign and symptoms of pertussis and lack of laboratory facilities to confirm the diagnoses. In addition, the reported high pentavalent three coverages may make the pertussis the least prioritized cause of the outbreak by the RRT. Furthermore, since pertussis is not included in the list of 21 surveillance targeted reportable diseases, the failure of management may be explained with lack of clear guidelines. Despite this, with the support of the Ethiopian Public Health Institute, the World Health Organization and USAID funded development partners, the district health office confirmed the cause of the outbreak and took corrective actions in the management of presumed and confirmed pertussis cases [32]. The mass azithromycin prophylactic treatment was delayed, which meant that suspected cases and their contacts could not be halted from pertussis transmission in the community [33]. In addition, administering treatments to all inhabitants of the affected 14 villages is not in line with global recommendations [34]. On the other hand, the reported post outbreak interventions and follow-up for two months was in line with the national PHEM guidelines.

### Strength and limitations of the study

The strength of this study is in its efforts to link the facility-based data quality with community-based immunization coverages. However, like many cross-sectional studies, it has known limitations to claim causalities. In addition, the investigators use of caretakers’ oral report on vaccination status might recall biases.

## Conclusions

Immunization data completeness was found to be optimal. However, in the study area, the accuracy, consistency, timeliness, and quality of the monitoring system were found to be suboptimal. Therefore, poor data quality has led to incorrect decision making during the reported pertussis outbreak management. Enhancing the capacity of healthcare providers on planning, recording, archiving, and reporting, analyzing, and using immunization data for evidence-based decision making is recommended. Improving the availability of recording and reporting tools is also likely to enhance the data accuracy and completeness of the community health information system. In addition, digitalizing the routine health information system requirements will improve the timelines and completeness of health data. Adapting pertussis outbreak management guidelines and conducting regular data quality assessments with knowledge sharing events to all stakeholders is recommended.

## Supplementary Information

**Additional file 1.** The Ethiopian vaccination schedule of routine EPI, September 2019

**Additional file 2.** Question on Quality for district level

**Additional file 3.** Question on Quality health facility level

## Data Availability

The datasets used and/or analyzed during the current study are available from the corresponding author on reasonable request.

## Abbreviations

DQS: Data Quality Self-Assessment
EPI: Expanded Program of Immunization
FMOH: Federal Ministry of Health
HEW: Health Extension Workers
HMIS: Health Management Information System
HP: Health Post
IRB: Institutional Review Board
MCV: Measles Containing Vaccine
MDG: Millennium Development Goal
PHCU: Primary Health Care Unit
QI: Quality Index
SDGs: Sustainable Development Goal
SNNPR: Southern Nations, Nationalities, and Peoples' Region
RRT: Rapid Response Team
UNICEF: United Nations Children's Fund
USAID: United States Agency for International Development
VPD: Vaccine Preventable Disease
WHO: World Health Organization
