# Supplementary material for: Immunization data quality and decision making in pertussis outbreak management in southern Ethiopia: a cross sectional study
Source: Arch Public Health. 2022 Feb 14;80:49. doi: 10.1186/s13690-022-00805-6 (PMC8842801; doi:10.1186/s13690-022-00805-6)
Supplement: Supplementary file 1 — Additional file 1. The Ethiopian vaccination schedule of routine EPI, September 2019 [file 13690_2022_805_MOESM1_ESM.docx]

| **Additional file 1:-The Ethiopian vaccination schedule of routine EPI, September 2019** | | |
| --- | --- | --- |
| Age | Visit | Antigen |
| At birth | 1 | BCG, OPV­0 |
| 6 weeks | 2 | DTP-HepB1-Hib1, OPV1, PCV1, Rota1 |
| 10 weeks | 3 | DTP-HepB2-Hib2, OPV2, PCV2, Rota2 |
| 14 weeks | 4 | DTP-HepB3-Hb3, OPV3, PCV3, IPV |
| 9 months | 5 | Measles1 (MCV1) |
| 15 months | 6 | Measles2 (MCV2) |

**NB:** BCG: Bacillus Calmette-Guerin; HepB: Hepatitis B; OPV: Oral Poliovirus Vaccine; DTP: Diphtheria, Tetanus, Pertussis; Pentavalent: Diphtheria, Pertussis, Tetanus, Hepatitis B, and Hemophilus Influenzae type b; IPV: Injectable Polio Vaccine; and MCV: Measles Containing Vaccine.
