# Supplementary material for: Immunization data quality and decision making in pertussis outbreak management in southern Ethiopia: a cross sectional study
Source: Arch Public Health. 2022 Feb 14;80:49. doi: 10.1186/s13690-022-00805-6 (PMC8842801; doi:10.1186/s13690-022-00805-6)
Supplement: Supplementary file 2 — Additional file 2. Question on Quality for district level [file 13690_2022_805_MOESM2_ESM.docx]

Additional file 2: Question on Quality for district level

| **EPI Data Quality ASSESSMENT**  **Question on Quality (QQ) district Level** | | |
| --- | --- | --- |
| **Question** | | **Coding Categories** |
| **Part I: Basic Information** | | |
| Date of assessment | __ __/__ __/__ __ __ __(DD/MM/YYYY) | |
| Organization name |  | |

1. Recording component (district)

| Ser no. | Question | Designed to find out | Weight 1 to 3 |
| --- | --- | --- | --- |
| 1 | Are vaccine receipts and issues recorded in a vaccine ledger? | To assess appropriate record-keeping of vaccine receipts and issues. |  |
| 2 | Does the district manager know the annual vaccine requirements for the district? |  |  |
| 3 | Is the lot number/batch number and expiry date of the vaccine recorded? |  |  |
| 4 | Is the current ledger up to date for a given vaccine (or vitamin A)? |  |  |
| 5 | Is there a log of syringe supply and delivery to HC/HP up to date? | Is the stock available identical to the quantity recorded in the register (count). |  |
| 6 | Are district staff aware of standard operating procedures to record a severe adverse event following immunization (AEFI)? |  |  |
| 7 | Do the district’s reports (found at district level) have at least one date stamped or written on them? | Determine what proportion of correct HC/HP reports you would need to answer “Yes”. Define for which period (e.g. for the previous year)Define the date significance: the date the report was signed the date of receipt at district level (stamped or written on it by the district office) |  |
| 8 | Are the district reports (that are sent to more central levels) correctly filled in? | To select a number of fields to be checked in all district reports and check whether these have been correctly filled in. |  |

1. Archiving component (district)

| Ser no. | Question | Designed to find out | Weight 1 to 3 |
| --- | --- | --- | --- |
| 9 | Is there a separate file or sub-file for each HC/HP and are the reports inside filed by date? | Storage should facilitate retrieval and monitoring (and be well organized). |  |
| 10 | Have all HC/HP data from the previous month been processed? |  |  |
| 11 | Are supervisory reports available? |  |  |
| 12 | Are copies of the last feedback to the health facilities easily available? |  |  |
| 13 | Can copies of all district reports (that were sent to more central levels) be found? |  |  |

1. Computerized archiving (district)

| Ser no. | Question | Designed to find out | Weight 1 to 3 |
| --- | --- | --- | --- |
| 14 | If the district is computerized is the last date of backup within one week?(look at the date the file was created on the diskette) | Check diskette for last saved date; look at the file creation date. |  |
| 15 | Can the official immunization tabulations for the previous year be reproduced from an archived electronic file? | To check official immunization tabulations = final summary of previous year data. |  |
| 16 | If more than one computer has immunization data, is there either or a written, well-organized method of data transfer? |  |  |
| 17 | (If yes, 17 Is the date of printing /production on every tabulation/if the data is archived, is there a date showing when the archived |  |  |

1. Reporting component (district)

| Ser no. | Question | Designed to find out | Weight 1 to 3 |
| --- | --- | --- | --- |
| 18 | Have the district reports of the last year month been sent on time? |  |  |
| 19 | Is the procedure for dealing with late reports known and applied? |  |  |
| 20 | Did all the monthly (quarterly) reports from the HC/HPs use the same form/format for the current year? |  |  |
| 21 | Is there a system for investigation of individual reports of adverse events following immunization (AEFI) from the district to the higher level functioning/operational? | Serious An adverse event following immunizations (AEFIs) should be rapidly reported and investigated.  Investigators should be looking for any evidence of programmatic error that must be rapidly corrected and/or rumors that cause problems. |  |
| 22 | Did all the visited HC/HPs report adequate supply of administrative forms tally sheets/reporting forms/health cards? |  |  |

1. Demographic information component (district)

| Ser no. | Question | Designed to find out | Weight 1 to 3 |
| --- | --- | --- | --- |
| 23 | Is the district denominator for immunization of infants and pregnant women (and school children, if applicable) known? | Known: the interviewed senior staff member should be able to tell (without looking) approximately how many infants the district contained and how the figure was calculated (if relevant). |  |
| 24 | Is there a district map of the catchment area showing HC/HPs and providing immunization strategies (fixed, outreach, mobile)? | Ideally, the map should include denominator, target, type of strategy. |  |
| 25 | Is the proportion of infants per strategy-type known for the district? | Usually fixed – outreach – mobile team, etc. This should be used in a district micro plan. |  |
| 26 | Has the same denominator for child immunization been used on different tabulations, reports, charts, tables, etc? | Indicate for which year. |  |
| 27 | Are the denominators used in the current year different from the denominators in the previous year? | Should be different from previous year. |  |
| 28 | For the previous year, is the district denominator value (for child immunizations) found at the district the same as that used at national level? |  |  |
| 29 | Is the denominator established independently? | The denominator should be established independently |  |
| 30 | Are the denominators of each HC/HP available for the previous year? | Answer “Yes” if available.  Totals should add up to the district total. |  |
| 31 | For the previous year, has only one denominator value (check at least total population) been seen in all health projects/ programmes? | Check with various initiatives (e.g. polio, nutrition, malaria) whether the denominator is consistent at district level. |  |

1. Core outputs/analysis component (district)

| Ser no. | Question | Designed to find out | Weight 1 to 3 |
| --- | --- | --- | --- |
| 32 | Is there a target number of children that the district strives to vaccinate during a calendar year or reporting period? |  |  |
| 33 | Is there a chart or table of immunization coverage by report period for the current year (monitoring chart)?  Is it on display? Is it UP TO DATE? Does it cover all antigens? |  |  |
| 34 | Is the completeness of the immunization reporting from HC/HP recorded and monitored at district level? | District staff should be able to describe what percentage of HC/HP reports was received on time, received but not on time, and not received at all during the previous year or the last months. |  |
| 35 | Does the district record and monitor timeliness for HC/HP immunization reporting? | District staff should be able to say (based on printed information) what percentage of HC/HP reports was received on time during the previous year or the last months. |  |
| 36 | Is the drop-out rate monitored?  Discuss the importance and reasons for dropouts. Are there managerial practices that could be changed to reduce the drop-out rate? |  |  |
| 37 | Is there monitoring of HC/HP/district vaccine wastage? | Discuss the importance and reasons for wastage. |  |
| 38 | Is there a graph by month of the incidence of vaccine-preventable diseases – broken down by VPD?  How do these data correspond to coverage data (i.e. more cases in with poor coverage).  When was the last VPD outbreak? Was it investigated?  Why did it occur? |  |  |
| 39 | Is an up-to-date chart/table of the completeness of the current year’s immunization data available? | Completeness = reports received or not received from the HC/HPs. (Here the score 0/1 is only for completeness.) |  |
| 40 | Is the HC/HP performance monitored at the district level? | Monitoring of HC/HPs: graph/figures showing how all current year. |  |
| 41 | Are supervision activities monitored? | A written schedule of supervision that includes visiting every HC/HP of time. |  |
| 42 | Has the district selected an indicator ? |  |  |

1. Evidence of using data for action (district)

| Ser no. | Question | Designed to find out | Weight 1 to 3 |
| --- | --- | --- | --- |
| 43 | Is there an analysis of HC/HP data performed regularly with HC/HP staff?  Analysis can be done within supervisory visits, meetings at district level, etc. Explore the quality of analysis as well as the exhaustiveness of the HC/HPs said to be analyzed: none of them should be left out. |  |  |
| 44 | Do you send regular monthly written feedback to the HC/HPs? |  |  |
| 45 | Are areas of low access identified and evidence of action taken to deal with it?  Discuss the importance and reasons for low access. How do the three strategies(fixed site, outreach and mobile teams) relate to the issue of access in the district? |  |  |
| 46 | Have reasons for any high drop-out been identified, and are there plans/actions to deal with it?  Are there managerial practices that could be changed to reduce the dropout rate? |  |  |
| 47 | Is there monitoring of HC/HP vaccine stock-outs?  (A stock-out is an interruption in vaccine supply [for any vaccine].) | The manager should be able to say (based on written information) whether any HC/HP has encountered a vaccine stock-out. If no vaccine stock-out is reported, ensure that the monitoring is possible and is being implemented. Staff should be monitoring the level of reserve stocks and taking action stock goes below a specified reserve level. |  |
| 48 | Are there problems with completeness and timeliness Are the late or incomplete reports usually from the same HC/HPs.  What was done to follow them up?  What other actions were taken to encourage/induce? |  |  |
| 49 | Are the recommendations made for the last in subsequent visits? |  |  |
| 50 | Has the monitoring of the selected immunization during the last 12 months? |  |  |
| 51 | Are surveillance and coverage then followed up to understand? |  |  |

Question on quality health facility level

| **EPI Data Quality ASSESSMENT**  **Question on Quality (QQ) health facility level** | | |
| --- | --- | --- |
| **Question** | | **Coding Categories** |
| **Part I: Basic Information** | | |
| Date of assessment | __ __/__ __/__ __ __ __(DD/MM/YYYY) | |
| Organization name | Health Center(HC)/ Health Post (HP) | |

1. Recording component (HC/HP)

| Ser no. | Question | Designed to find out | Weight 1 to 3 |
| --- | --- | --- | --- |
| 1 | Are there tally sheets for infant vaccinations on the desk (or easily available) and do they have entries for the last immunization day? | The main concern is evidence of use of availability (official form) and tally sheet. |  |
| 2 | Are registers used for recording individual information about child immunizations? | Each HC/HP should have a book or register where each immunization history can be registered and traced back. |  |
| 3 | Can a child’s vaccination history be easily and rapidly retrieved in the registers?  A new dose should not be entered as a complete new entry but entered in the location where previous doses have been entered. | Score 0 if the register issued as a new entry for any immunization. |  |
| 4 | Are registers (or pre-printed forms) used for recording individual information about women’s TT immunizations? | There may be registers or health cards if cards kept in HC/HP. |  |
| 5 | Observe at least five vaccinations: Were all vaccinations well registered on the child health card/tally sheet/ |  |  |
| 6 | Are individual immunization records used, updated and given to caretaker at the time of the immunization visit? | Blank immunization cards should be available in the HC/HP. |  |
| 7 | Are vaccine receipts recorded in a vaccine ledger, stock card, bin card, good receiving and issuing notes? | Check against available stock (count doses in the refrigerator) |  |
| 8 | Ask the child’s caretaker:  Do you know the expected date? | Find out whether the expected dates are known |  |
| 9 | Is the ledger /stock card/bin card/good receiving and issuing notes/ up to date for all vaccines and/vaccine?  Up to date = all receipts and issues recorded immediately. | Check against stock (in the refrigerator). |  |
| 10 | Is the receipt of a selected vaccine in the ledger complete for the entire year? |  |  |
| 11 | Is there a log (vaccine ledger/stock card) for receipt/issuing of syringes supplied (AD/non-AD reconstitution syringes)? | Can perform a stock check. |  |
| 12 | Does the HC/HP record vaccine batch-number and expiry date? |  |  |
| 13 | Are all individual recording forms available for the entire previous year? | recording form = tally sheet or register. |  |
| 14 | Did every person doing the child immunization card exercise get a perfect score for:PENTA1, PENTA3, measles? | Need to define how the scoring will be if a perfect score is not obtained. |  |
| 15 | Is the cold chain temperature monitoring chart completed daily? | Check the chart and compare the latest reported temperature with the actual temperature in the refrigerator. |  |

1. Report component (HC/HP)

| Ser no. | Question | Designed to find out | Weight 1 to 3 |
| --- | --- | --- | --- |
| 16 | Have all reports for the previous year been signed by the officer-in-charge or officer authorized to submit the HC/HP report? | Score for example: If >50% reports are signed score “Yes”. If <100% discuss with the HC/HP why some have not been signed. |  |
| 17 | Does each report from the previous year have at least one date stamped or written on it by the HC/HP – either as “signed date” or “compiled date”? | If >50% reports are signed score “Yes”. If <100% discuss with the HC/HP why some have no data stamped or written in. This can be answered at district level. |  |
| 18 | Are the HC/HP reports correctly filled in? | Select a number of fields to be checked in all HC/HP reports and check whether these have been filled in correctly. |  |
| 19 | Are health staff aware of standard operating procedures and the necessary forms to complete if there is a report of a severe AEFI? | Ask health staff what is supposed to be done if a child becomes severely ill or dies after a vaccination.  Ask to see any forms that are to be used. |  |
| 20 | Are the HC/HP reports completely filled in? | Select a number of fields to be checked in all HC/HP reports from the previous year and check whether these have been filled in. |  |

C. Archiving component (HC/HP)

| Ser no. | Question | Designed to find out | Weight 1 to 3 |
| --- | --- | --- | --- |
| 21 | Can copies of all previous reports from this HC/HP be found in the HC/HP? | For current and previous year. |  |
| 22 | Is there one location where the previous immunization reports and recording forms are stored? |  |  |
| 23 | Are the reports of the HC/HP organized in a file by date? | The main concern is that the reports are easily retrievable. |  |
| 24 | Are HC/HP reports available for the entire year? |  |  |
| 25 | Are the child registers available for all periods of the previous year? |  |  |
| 26 | Can all tally sheets covering the previous year be found? |  |  |
| 27 | Are registers for TT vaccinations to pregnant women available for the entire previous year? |  |  |
| 28 | Is the latest feedback on data from district easily available? |  |  |

1. Demographic information component (HC/HP)

| Ser no. | Question | Designed to find out | Weight 1 to 3 |
| --- | --- | --- | --- |
| 29 | Does the HC/HP have data on the number of infants born in its catchment area? | The number of births should be different from the previous year. Discuss if there is a difference with the denominator available at more central level. Discuss ways to collect denominator information from community (e.g. birth register), data from national immunization days (NIDs), or other sources. Discuss if the target was set up by the district or HC/HP level. |  |
| 30 | Does the HC/HP have a target set on the number of children that should be vaccinated during the calendar year? | Discuss how realistic the value is. |  |
| 31 | Does the HC/HP have a system that allows the collection of information on new births in the community? | This may include community health workers, traditional birth attendants, outreach clinics, etc.  A system means (a) organized way to collect the information in every village/community and (b) a written track available at the HC/HP. |  |
| 32 | Does the HC/HP have a target by type of strategy (fixed/outreach/mobile) with a map showing the catchment area by strategy including the outreach villages? |  |  |

1. Core outputs/analysis (HC/HP)

| Ser no. | Question | Designed to find out | Weight 1 to 3 |
| --- | --- | --- | --- |
| 33 | Does the HC/HP have a (target) number of children that it strives to vaccinate during a calendar year or a reporting period? |  |  |
| 34 | Is there a mechanism in place to track defaulters? | Can be an appropriate use of a correctly filled register, tickler file, etc.  When was the last time a child was followed up ? |  |
| 35 | Does the HC/HP have achievements split by type of strategy – fixed/outreach/mobile? | It is important is to know the proportion of numbers actually reached by each strategy. |  |
| 36 | Does the HC/HP have an up-to-date chart or table (preferably on display) showing the number of vaccinations by report period for the current year? | Monitoring coverage chart must be UP TO DATE. |  |
| 37 | Is there a monthly chart/graph of VPD cases (broken down by VPD)?  How do these data correspond to coverage data (i.e. more cases in areas with poor coverage)? | When was the last VPD outbreak? Was it investigated?  Why did it occur? |  |
| 38 | Does the HC/HP monitor drop-out rate? | Preferably on display with the same monitoring chart as the coverage but score 1 if the health worker can tell you the drop-out rate Discuss the importance and reasons for dropouts. |  |
| 39 | Does the HC/HP monitor vaccine wastage? | Discuss the reasons for wastage and any ways it might Discuss whether the health worker knows how much is and how it can be calculated. |  |

1. Evidence of using data for action component (HC/HP)

| Ser no. | Question | Designed to find out | Weight 1 to 3 |
| --- | --- | --- | --- |
| 40 | Is there a mechanism in place to track defaulters or vaccine doses that are due? | Check how the HC/HP can know when a child should return for a vaccine dose (e.g. PENTA, Hib, HepB, polio, measles or yellow fever vaccine dose). |  |
| 41 | Is there a map showing the catchment area, including the outreach villages? | This ideally shows strategy type. |  |
| 42 | Are areas of low access identified and is there evidence of actions taken to deal with this? | If there is low access (evidenced by low BCG or PENTA1 coverage), how does it relate to the effectiveness of the three strategies (fixed site, outreach and/or mobile teams |  |
| 43 | Have reasons for any high drop-out been identified; are there plans/actions to deal with this? | Are there any managerial practices that can be changed? |  |
| 44 | Have actions been taken on the last feedback from the district? |  |  |
| 45 | Is there interaction with the community regarding immunization?  Ask for information on “what” and “when”? | Are health staff actively involved in any community committees or meetings on health, investigations of outbreaks or any rumors of An adverse event following immunizations (AEFIs), etc? |  |
