# Supplementary material for: Immunization data quality and decision making in pertussis outbreak management in southern Ethiopia: a cross sectional study
Source: Arch Public Health. 2022 Feb 14;80:49. doi: 10.1186/s13690-022-00805-6 (PMC8842801; doi:10.1186/s13690-022-00805-6)
Supplement: Supplementary file 3 — Additional file 3. Question on Quality health facility level [file 13690_2022_805_MOESM3_ESM.docx]

## Additional file 3: Data Quality and outbreak management assessment tools

## For Secondary Data Review and interview

1. **Heath Post Questionnaire Code _______**

**the capacity of the health system to identify, manage, and follow the responses to vaccine preventable disease outbreak**

**SECTION-I: Identification**

Name of Zone _________________ Name of Woreda ________________________________

Health Post ___________ Date of the assessment (DD/MM/YYYY) ____________

Data collector name and signature ___________________________________________________

Supervisor name and signature ___________________________________________________

## **General Information**

- - Catchment total population ___________
  - Numbers of Under one children (< 11 months) ___________
  - Number of HEWs workers in the HP ___________

## **Availability of data collection tools and supplies**

| **Registry and tool** | **Available** | **Not available** | **Available but not used** | **Remark** |
| --- | --- | --- | --- | --- |
| Registers/family folder |  |  |  |  |
| Tally sheets, |  |  |  |  |
| Immunization monitoring charts |  |  |  |  |
| Monthly immunization reports (HMIS) |  |  |  |  |
| Vaccine stock record, |  |  |  |  |
| Vaccine Requisition Format, |  |  |  |  |
| Temperature recording sheet, if fridge is available |  |  |  |  |
| Microplanning |  |  |  |  |
| Defaulter tracking system |  |  |  |  |

## **Availability of vaccines and supply**

- 1. Did you face any shortage of vaccines and supplies in last six months?
     - Vaccines 1. Yes 2. No
     - Dilutants 1. Yes 2. No
     - Syringes 1. Yes 2. No
     - Functional refrigerators 1. Yes 2. No
  2. Is there any stockout of vaccines, dilutants and syringes during the time of visit---------- 1. Yes 2. No

If yes, please list them _____________________________________________

- 1. If there is no refrigerator how they are managing immunization services ___________________

____________________________________________________________________

## **Immunization practice**

- 1. Is there daily immunization service at the health post -------- 1. Yes 2. No
     1. If No, what is the possible reason _______________________________________
  2. Do you have outreach sessions ------- 1. Yes 2. No
     1. If yes, do you have outreach session plan ---------- 1. Yes 2.No
     2. If yes, proportion of outreach sessions implemented in the last 6 months _______
  3. Is there any hard to reach areas in the catchment area-------- 1. Yes 2.No
     1. If yes, how far from the health post (Km) ________
     2. How can you access those target group? ___________________________________

## **Immunization data completeness and consistency with different source**

- 1. Is the registration book filled and complete? 1. Yes 2. No
  2. Is the recording kept updated with new eligible children? (i.e. are new infants reaching 6 months recorded and added monthly) 1. Yes 2. No

| SN | Antigen | 2011 6 months Performance achievement by year | | | |
| --- | --- | --- | --- | --- | --- |
|  |  | Register/ Family folder | Tally sheet | Month report | Monitoring chart |
| 1 | Penta 1 |  |  |  |  |
| 2 | Penta 3 |  |  |  |  |
| 3 | Pent DOR |  |  |  |  |
| 5 | Measles1 |  |  |  |  |

- 1. Consistency of HP immunization data at different level

| **Report Level** | **Penta 1 coverage** | **Penta 3 coverage** | **Measles coverage** | **FIC coverage** |
| --- | --- | --- | --- | --- |
| **Name of the health post_____________________________** | | | | |
| Health post report |  |  |  |  |
| Health center report copy |  |  |  |  |
| Woreda level report copy |  |  |  |  |
| **Name of the health post_____________________________** | | | | |
| Health post report |  |  |  |  |
| Health center report copy |  |  |  |  |
| Woreda level report copy |  |  |  |  |
| **Name of the health post_____________________________** | | | | |
| Health post report |  |  |  |  |
| Health center report copy |  |  |  |  |
| Woreda level report copy |  |  |  |  |
| **Name of the health post_____________________________** | | | | |
| Health post report |  |  |  |  |
| Health center report copy |  |  |  |  |
| Woreda level report copy |  |  |  |  |

## **Immunization data use for action**

- 1. Did they fill the immunization monitoring chart properly? a) Yes b) No
  2. If yes, did they identify poorly covered areas (accessibility and utilization) a) Yes b) no
  3. If yes, did they design special strategy to address those areas a) yes b) no
  4. If no, would you please list those major challenges___________________________

## **Reported vaccine preventable diseases and investigation status in the previous half year**

|  | **Vaccine Preventable Diseases** | **Not identified (Yes/No)** | **Reported**  **(Yes/No)** | **Investigated and confirmed**  **(Yes/No)** | **Responded**  **(Yes/No)** | **Followed up and recovery**  **(Yes/No)** |
| --- | --- | --- | --- | --- | --- | --- |
| 1 | Measles |  |  |  |  |  |
| 2 | Pertussis |  |  |  |  |  |
| 3 | Yellow fever |  |  |  |  |  |
| 4 | Others |  |  |  |  |  |

## NB: If it is not identified, fill no in the consecutive column

## **Capacity enhancement and performance monitoring**

- 1. Are there EPI trained health care workers in the past two years a) yes b) no
- If yes, type of the training provided _________________________________
- If yes, number of HCWs received the training ___________________________
  1. How frequent do you get EPI supervision support from different levels

1. Monthly
2. Quarterly
3. Biannual
4. Based on convenience schedule

Major findings of the supportive supervision _____________________________________

1. **Health Center Questionnaire Code _______**

**SECTION-I: Identification**

Name of Zone _________________ Name of Woreda ________________________________

Health center ___________ Date of the assessment (DD/MM/YYYY) ____________

Data collector name and signature ___________________________________________________

Supervisor name and signature ___________________________________________________

## **General Information**

- - Catchment total population ___________
  - Numbers of Under one children (< 11 months) ___________
  - Number of health posts _______________________
  - Number of HEWs workers in the HP ___________
  - Number of HCW in the PHCUs __________________

## **Availability of data collection tools and supplies**

| **Registry and tool** | **Available** | **Not available** | **Available but not used** | **Remark** |
| --- | --- | --- | --- | --- |
| Registers |  |  |  |  |
| Tally sheets, |  |  |  |  |
| Immunization monitoring charts |  |  |  |  |
| Monthly immunization reports (HMIS) |  |  |  |  |
| Vaccine stock record, |  |  |  |  |
| Vaccine Requisition Format, |  |  |  |  |
| Temperature recording sheet |  |  |  |  |
| Microplanning |  |  |  |  |
| Defaulter tracking system |  |  |  |  |
| Supervision checklists reports and feedbacks, |  |  |  |  |

## **Availability of vaccines and supply**

- 1. Did you face any shortage of vaccines and supplies in last six months?
     - Vaccines 1. Yes 2. No
     - Dilutants 1. Yes 2. No
     - Syringes 1. Yes 2. No
     - Functional refrigerators 1. Yes 2. No
  2. Is there any stockout of vaccines and dilutants during the time of visit---------- Yes/ No

If yes, please list them _____________________________________________

## **Immunization practice**

- 1. Is there daily immunization service at the health center -------- Yes /No
     1. If No, what is the possible reason _______________________________________
  2. Do you have outreach sessions ------- Yes/No
     1. If yes, do you have outreach session plan ---------- Yes/No
     2. If yes, proportion of outreach sessions implemented in the last 6 months _________
  3. Is there any hard to reach areas in the catchment area-------- Yes/No
     1. If yes, how far from the health facilities ________ (Km)
     2. How can you access those target group? ___________________________________

## **Immunization data completeness and consistency with different source**

- 1. Is the registration book filled and complete? 1. Yes 2. No
  2. Is the recording kept updated with new eligible children? (i.e. are new infants reaching 6 months recorded and added monthly) 1. Yes 2. No

| SN | Antigen | 2011 6 months Performance achievement by year | | | |
| --- | --- | --- | --- | --- | --- |
|  |  | Register | Tally sheet | Month report | Monitoring chart |
| 1 | Penta 1 |  |  |  |  |
| 2 | Penta 3 |  |  |  |  |
| 3 | Pent DOR |  |  |  |  |
| 5 | Measles1 |  |  |  |  |

## **Immunization data use for action**

- 1. Did they fill the immunization monitoring chart properly? a) Yes b) No
  2. If yes, did they identify poorly covered areas (accessibility and utilization) a) Yes b) no
  3. If yes, did they design special strategy to address those areas a) yes b) no
  4. If no, would you please list those major challenges___________________________

## **Reported vaccine preventable diseases and investigation status in the previous half year**

| **SN** | **Vaccine Preventable Diseases** | **Not identified (Yes/No)** | **Reported**  **(Yes/No)** | **Investigated and confirmed**  **(Yes/No)** | **Responded**  **(Yes/No)** | **Followed up and recovery**  **(Yes/No)** |
| --- | --- | --- | --- | --- | --- | --- |
| 1 | Measles |  |  |  |  |  |
| 2 | Pertussis |  |  |  |  |  |
| 3 | Yellow fever |  |  |  |  |  |
| 4 | Measles |  |  |  |  |  |
| 5 | Others |  |  |  |  |  |

## **Capacity enhancement and performance monitoring**

- 1. Are there EPI trained health care workers in the past two years a) yes b) no
- If yes, type of the training provided _________________________________
- If yes, number of HCWs received the training ___________________________
  1. How frequent do you get EPI supervision support from different levels

1. Monthly
2. Quarterly
3. Biannual
4. Based on convenience schedule

Major findings of the supportive supervision _____________________________________

1. **Woreda Health Office Questionnaire Code _______**

**SECTION-I: Identification**

Name of Zone _________________ Name of Woreda ________________________________

Date of the assessment (DD/MM/YYYY) ____________

Data collector name and signature ___________________________________________________

Supervisor name and signature ___________________________________________________

## **General Information**

- - Catchment total population ___________
  - Numbers of Under one children (< 11 months) ___________
  - Number of health Centers _______________________
  - Number of HCW in the PHCUs __________________
  - Number of health posts _______________________
  - Number of HEWs workers in the HP ___________

## **Availability of data collection tools and supplies**

| **Registry and tool** | **Available** | **Not available** | **Available but not used** | **Remark** |
| --- | --- | --- | --- | --- |
| Registers |  |  | NA |  |
| Tally sheets, |  |  | NA |  |
| Immunization monitoring charts |  |  |  |  |
| Monthly immunization reports (HMIS) |  |  |  |  |
| Vaccine stock record, |  |  |  |  |
| Vaccine Requisition Format, |  |  |  |  |
| Temperature recording sheet |  |  |  |  |
| Aggregated microplan |  |  |  |  |
| Supervision checklists reports and feedbacks, |  |  |  |  |

## **Availability of vaccines and supply**

- 1. Did you face any shortage of vaccines and supplies in last six months?
     - Vaccines 1. Yes 2. No
     - Diluentants 1. Yes 2. No
     - Syringes 1. Yes 2. No
     - Functional refrigerators 1. Yes 2. No
  2. Is there any stockout of vaccines and diluentants during the time of visit---------- Yes/ No

If yes, please list them _____________________________________________

## **Immunization data completeness and consistency with different source**

| SN | Antigen | 2011 6 months Performance achievement by year | | |
| --- | --- | --- | --- | --- |
|  |  | Monthly report received from PHCUs | HMIS | Monitoring chart |
| 1 | Penta 1 |  |  |  |
| 2 | Penta 3 |  |  |  |
| 3 | Pent DOR |  |  |  |
| 5 | Measles1 |  |  |  |

## **Immunization data use for action**

- 1. Did they fill the immunization monitoring chart properly? a) Yes b) No
  2. If yes, did they identify poorly covered areas (accessibility and utilization) a) Yes b) no
  3. If yes, did they design special strategy to address those areas a) yes b) no
  4. If no, would you please list those major challenges___________________________

## **Reported vaccine preventable diseases and investigation status in the previous half year**

| **SN** | **Vaccine Preventable Diseases** | **Not identified (Yes/No)** | **Reported**  **(Yes/No)** | **Investigated and confirmed**  **(Yes/No)** | **Responded**  **(Yes/No)** | **Followed up and recovery**  **(Yes/No)** |
| --- | --- | --- | --- | --- | --- | --- |
| 1 | Measles |  |  |  |  |  |
| 2 | Pertussis |  |  |  |  |  |
| 3 | Yellow fever |  |  |  |  |  |
| 4 | Others |  |  |  |  |  |

## **Capacity enhancement and performance monitoring**

- 1. Are there EPI trained health care workers in the past two years a) yes b) no
- If yes, type of the training provided _________________________________
- If yes, number of HCWs received the training ___________________________
  1. How frequent do you get EPI supervision support from different levels

1. Monthly
2. Quarterly
3. Biannual
4. Based on convenience schedule

Major findings of the supportive supervision _____________________________________

1. **Key informant interview Guide:**

**For Woreda Health Office/Public Health Emergency Management team/**

**Key informant interview Guide to assess the capacity of the health system to identify, manage, and follow the responses to vaccine preventable disease outbreak**

Questionnaire Code ___________

**How did you control the pertussis epidemic in your district?**

**Guiding questions**

**Governance, epidemic response multi-sectoral coordination and community engagement**

1. Does the woreda have functional rapid response team ? **Probe**: with clear terms of reference and representation of key stakeholders, and key activities implemented by the committee?
2. Does the woreda established sub committees in the Woreda? **Probe**: is it possible to mention their names? Do they have clear terms of reference?
3. Does the woreda have epidemic response coordination committee? **Probe**: members of the committee, how often held meetings during the outbreak? Provide feedback or directions to the response team? Developed concept notes to access resources, communicate partners and respective government system to get support
4. Do the woreda have risk communication, social mobilization and advocacy team to enhance the awareness of the community about the outbreak (transmission, prevention, treatment ….)? **Probe**: what kind of communication means they used? Community meetings, distribution of IEC/BCC materials, health education at the health facilities, use of local media
5. How was the engagement of communities (community representatives, religious leaders, school communities) during the epidemic? **Probe**: there roles and responsibilities,

**Surveillance and laboratory Confirmation**

1. How did you confirm the existence of the outbreak? **Probe**: with possible sign and symptoms, laboratory confirmation,
2. How was the surveillance system during the outbreak? **Probe**: developed standard case definitions and distributed to health facilities, early identification of cases, daily reporting from health facilities and communities, contact tracing, use of line list, provided feedback to the epidemic response team (trend, areas mostly affected, affected groups by their vaccination status, treatment outcome…)

**Case management**

1. How was the case management system during the outbreak? **Probe**: provided orientation to the health professionals, prepared and distributed case management guidelines, procured and distributed drugs and supplies, Was there any shortage of supplies and drugs? Referral system for further management of cases, Case fatality rate (number of deaths)

**Evaluation of the outbreak response**

1. Was there any outbreak response evaluation? **Probe**: Documentation of major challenges and lessons learned, facilitated review meeting
2. What was the major challenges of the epidemic response, recommendations to strengthen the health system?
3. Do you have any information that you want to share with us?

Thanks for your time
